# Prefrontal cortical hemodynamics and functional network organization during Tai Chi standing meditation: an fNIRS study

**DOI:** 10.3389/fnhum.2023.1294312

**Published:** 2023-10-25

**Authors:** Liping Qi, Guo-Liang Wang, Zhi-Hao Tian, Shuo Guan, Shu-Ye Yang, Yu-Long Yang, Li-Qing Liu, Yong-Zhong Lin

**Affiliations:** ^1^Faculty of Electronic Information and Electrical Engineering, Dalian University of Technology, Dalian, China; ^2^School of Physical Education and Health, Dalian University of Technology, Dalian, China; ^3^The Second Affiliated Hospital of Dalian Medical University, Dalian, China

**Keywords:** functional connectivity, general linear model, graph theory analysis, resting state, meditation, small-worldness, network hubs

## Abstract

**Introduction:**

Tai Chi standing meditation (Zhan Zhuang, also called pile standing) is characterized by meditation, deep breathing, and mental focus based on theories of traditional Chinese medicine. The purpose of the present study was to explore prefrontal cortical hemodynamics and the functional network organization associated with Tai Chi standing meditation by using functional near-infrared spectroscopy (fNIRS).

**Methods:**

Twenty-four channel fNIRS signals were recorded from 24 male Tai Chi Quan practitioners (54.71 ± 8.04 years) while standing at rest and standing during Tai Chi meditation. The general linear model and the SPM method were used to analyze the fNIRS signals. Pearson correlation was calculated to determine the functional connectivity between the prefrontal cortical sub-regions. The small world properties of the FC networks were then further analyzed based on graph theory.

**Results:**

During Tai Chi standing meditation, significantly higher concentrations of oxygenated hemoglobin were observed in bilateral dorsolateral prefrontal cortex (DLPFC), ventrolateral prefrontal cortex (VLPFC), frontal eye field (FEF), and pre-motor cortex (PMC) compared with the values measured during standing rest (*p* < 0.05). Simultaneously, significant decreases in deoxygenated hemoglobin concentration were observed in left VLPFC, right PMC and DLPFC during Tai Chi standing meditation than during standing rest (*p* < 0.05). Functional connectivity between the left and right PFC was also significantly stronger during the Tai Chi standing meditation (*p* < 0.05). The functional brain networks exhibited small-world architecture, and more network hubs located in DLPFC and VLPFC were identified during Tai Chi standing meditation than during standing rest.

**Discussion:**

These findings suggest that Tai Chi standing meditation introduces significant changes in the cortical blood flow and the brain functional network organization.

## 1. Introduction

Tai Chi Quan is a typical form of mind–body exercise, which is originated in ancient China. Tai Chi Quan differentiated into many schools in the process of development, such as Chen, Yang, Wu, and Sun. Each school has its own distinctive features, but the basic concepts of Tai Chi are the same. Tai Chi Quan has been practiced by the general public to promote physical and mental health (Abbott and Lavretsky, 2013; Chen et al., 2016). As a popular form of exercise throughout the world, Tai Chi Quan has drawn increasing research interest from international scientists. Tai Chi Quan consists of the coordination of gentle movement with diaphragmatic breathing to achieve tranquility of mind that involves cognitive training and movement meditation. Tai Chi Zhan Zhuang (standing meditation, also called pile standing) is a standing form of meditation widely known as “standing like a tree,” which is a static postural and breathing training. The goal of Tai Chi standing meditation (Zhan Zhuang) is learning to hold our body in a relaxed, extended, and open position. Tai Chi standing meditation has several benefits: improved posture and breathing, enhanced cognitive skills, sharper somatic awareness, and sustained mental attention (Miller and Taylor-Piliae, 2014).

The practice of meditation has proven to be associated with several physiological changes including the balance of sympathetic-parasympathetic activity (Telles et al., 2013), heart rate variability (Bashir et al., 2019; Kirk and Axelsen, 2020), blood pressure (Cernes and Zimlichman, 2015), and oxygen metabolism (Van Wijk et al., 2008). In particular, meditation improves cerebral blood flow and oxygenation levels of many areas of the brain (Luders et al., 2012). Meditation studies have also suggested that long-term meditation practice is associated with delayed aging of the brain (Luders et al., 2016). Previous neuroimaging studies have shown several regions to be active during meditation, including the prefrontal and orbital frontal cortex, right dorsal medial frontal lobe, cingulate gyrus and right sensorimotor cortex (Brefczynski-Lewis et al., 2007; Hasenkamp et al., 2012). An area of particular interest to this study is the dorsolateral prefrontal cortex (DLPFC) that has been shown to be active during several types of meditation, such as focused attention meditation (Brefczynski-Lewis et al., 2007; Hasenkamp et al., 2012), open-monitoring, and mindfulness meditation practices (Farb et al., 2007), as well as in response to affective stimuli in trained meditators (Farb et al., 2010). In addition, changes in DLPFC functional connectivity during meditation are well-documented, and in particular, an enhanced functional connectivity between the DLPFC and default mode networks in people with advanced meditation skills has been well-studied (Brewer et al., 2011; Creswell, 2016). However, there is limited study to investigate the brain activity during Tai Chi standing meditation (Zhan Zhuang) in a naturalistic environment.

In recent years, a compact portable non-invasive optical imaging tool, namely functional near infrared spectroscopy (fNIRS), has been used to measure the changes in oxy-hemoglobin and deoxy-hemoglobin from the cortical surface (Pinti et al., 2020). Moreover, wireless fNIRS equipment is safe, convenient, inexpensive, and less restrictive for the movement. Participants can thus move more naturally with fewer constraints (Pinti et al., 2020). Compared with other non-invasive brain detection techniques, such as fMRI and EEG, fNIRS can provide hemodynamic signals with high temporal sampling (up to 50 Hz) in a comfortable and natural situation, and relatively long periods of data acquisition can be tolerated much more easily by participants, which is suitable for monitoring brain activities of Tai Chi practitioners during standing meditation in a naturalistic environment. fNIRS has been used to investigate the changing concentration of oxy-, deoxy, and total hemoglobin during Qigong (Cheng et al., 2010) and Yoga meditation (Deepeshwar et al., 2014). The authors of those studies observed an increase in oxy-hemoglobin concentration in the PFC during meditation among the meditation practitioners. The objective of the present study was to investigate prefrontal cortex (PFC) activity and functional networks of Tai Chi practitioners during Tai Chi standing meditation in a natural environment by using fNIRS. The hypothesis was that Tai Chi standing meditation could increase the activation of PFC and functional connectivity between sub-regions of PFC in Tai Chi Quan practitioners.

## 2. Materials and methods

### 2.1. Participants

Twenty-four healthy male Tai Chi Quan practitioners were recruited from Wen County, China, the birthplace of Chen-style Tai Chi Quan. Local participants were enlisted by a community recruiter. Demographic characteristics of participants are shown in Table 1. The average age of the participants was 54.71 ± 8.04 years and their average Tai Chi practice experience was 17.46 ± 14.24 years. The participants had no cardio-cerebrovascular or respiratory diseases, history of mental illness, or brain trauma. The study was approved by the University Ethics Committee. Informed written consent was obtained from each participant before the test.

**Table 1:** Demographic characteristics of participants (mean ± SD, *n*%).

Characteristics	Mean ± SD or *n*%
Sample size	24
Age (years)	54.71 ± 8.04
Age range (years)	41–75
Height (cm)	170.4 ± 5.0
Weight (kg)	75.0 ± 8.8
BMI (kg/m^2^)	25.3 ± 2.3
Tai Chi practice experience (years)	17.46 ± 14.24 years
Range of practice experience (years)	2–60
Daily practice (hours)	1.5 ± 1.2
Range of daily practice (hours)	0.5–6
**Education**	
Post-secondary and higher education	25%
Others	75%
**Medication**	
No medication	75%
Antihypertensive drugs	25%
**Weekly alcohol consumption**	
0–≤25 g per week	38%
25–≤50 g per week	29%
250–≤350 g per week	8%
≥350 g per week	25%
**Smoking status**	
Non-smoker	87.5%
Very-light smoker (<6 cigarettes per day)	12.5%

### 2.2. Experimental procedure

**Figure 1:**
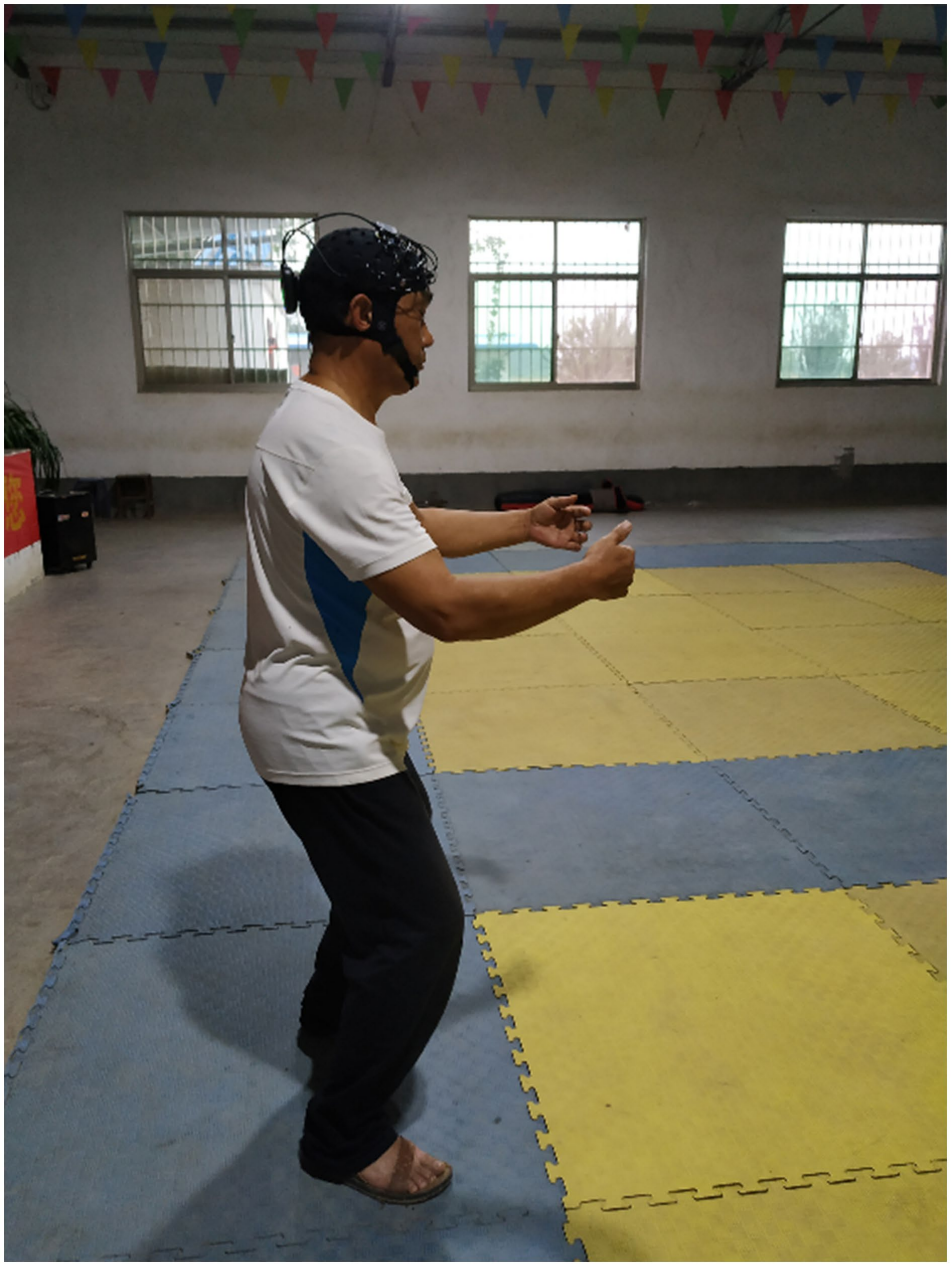
Zhan Zhuang is a standing meditation practice where one stands still with the eyes closed and in an upright posture, as if standing like a tree. Participants were totally relaxed and breathed deeply and naturally. Functional near infrared spectroscopic signals were recorded while standing at rest and standing during Tai Chi meditation.

The experiment consisted of two sessions: 10 min of standing rest followed by 10 min Tai Chi standing meditation. The order of standing rest and standing meditation sessions was counterbalanced, with a seated rest period of approximately 10 min interposed between them. During the Tai Chi standing meditation, participants stand in perfect balance with their eyes closed, feet flat on the ground at shoulder width and parallel to each other, knees bent slightly (high posture), with the relaxed cupped hands held in front of their chest (Figure 1). Participants were totally relaxed and breathed deeply and naturally, but as the same time they were focusing their internal energy with a tranquil and alert mind when they were in the meditative state.

### 2.3. Data acquisition

**Figure 2:**
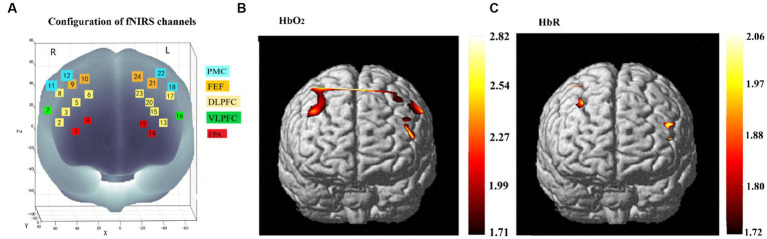
The configuration of fNIRS channels use in the present study **(A)** and cortical activation maps during the Tai Chi standing meditation in the group **(B,C)**. 8 transmitting and 10 receiving probes arranged alternately at an inter-probe distance of 3 cm, resulting in 24 channels per set. Estimated fNIRS channel locations are exhibited in MNI space. Five regions of interest in the PFC and motor cortex are indicated with colors and channel numbers. **(B)** Activation maps found by HbO_2_ during the Tai Chi standing meditation in the group. Activated regions are located in the PFC (bilateral DLPFC and VLPFC) and PMC (bilateral FEF and PMC). **(C)** Activation maps found by HbR during the Tai Chi standing meditation in the group. Activated regions found by the HbR were located in the left VLPFC, right PMC and DLPFC. Activations indicate regions with a greater response during the Tai Chi meditation state relative to the resting state (*p* < 0.05, expected Euler corrected). FPA, frontopolar area; DLPFC, dorsolateral prefrontal cortex; VLPFC, ventrolateral prefrontal; FEF, frontal eye field; PMC, pre-motor cortex.

A multichannel near-infrared spectroscopy system (Brite 24, Artinis Medical Systems, The Netherlands) was used to monitor the changes in hemodynamic response parameters of the prefrontal brain area while standing at rest and standing during Tai Chi meditation. The Brite 24 system includes 10 transmitting probes that emit near-infrared light and 8 receiving probes that detect near-infrared light. The distance between the transmitter and receiver is 3 cm. The differential path factor value was adjusted for each participant according to age. The Oxysoft software (Artinis Medical Systems, The Netherlands) was used to identify the MNI coordinates. The 24 measurement channels are distributed in the frontal prefrontal area (FPA), dorsolateral prefrontal cortex (DLPFC), ventrolateral prefrontal cortex (VLPFC), frontal lobe of the brain eye movement area (frontal eye field, FEF) and pre-motor cortex (pre-motor and supplementary motor cortex, PMC; Figure 2A).

### 2.4. Analysis of fNIRS data

#### 2.4.1. Brain activation analysis

Although fNIRS signals are robust to motion artifacts, relatively steady and high signal-to-noise ratio signals are still crucial for subsequent brain activation and networks analysis. The first 2 min fNIRS signals for each participant were removed because the initial signals were unstable due to the inadaptation of the participants to the measurement environments and/or the unachieved stationary state of the measurement equipment during both the resting state and meditation state. The 2 min to 4 min data were selected by visual inspection for 2 reasons: (1) to ensure the signals were relatively steady from each channel and participant; (2) to give participants enough time to reach a meditative state. Previous studies have shown that 2 min resting state fNIRS data are sufficient for imaging and functional networks analysis (Geng et al., 2017). The 2 min fNIRS signals chosen were then analyzed using the NIRS-SPM toolbox after an initial analysis based on the modified Beer–Lambert law (Delpy et al., 1988; Chul et al., 2009). The changes in oxygenated hemoglobin (HbO_2_) and deoxygenated hemoglobin (HbR) concentration were calculated into units of millimolar–millimeter (mM·mm). The HbO_2_ and HbR signals of each channel were preprocessed with a high-pass filter (1/60 Hz) to reduce the global trend and to improve the signal to noise ratio. The baseline drift was removed with a cut-off frequency of 0.04 Hz, and the heartbeat pulsation was filtered at 0.3 Hz. A general linear model (GLM) was used for the first-level analysis, and a comparison of channel and participant aspects from preprocessed time series data was performed for second-level random-effect group analysis using the SPM method (Penny and Holmes, 2006). Control of familywise errors in the expected value calculation was achieved by the Euler method.

#### 2.4.2. Functional connectivity and small world properties

Functional connectivity (FC) was analyzed by using the toolbox FC-NIRS (Xu et al., 2015). Each channel was defined as a node, and the connection between nodes were defined as an edge in the network. The edges were identified by the Pearson correlation. The Pearson correlation coefficients were calculated, and then the Fisher transformation was applied to transform the Pearson correlation coefficients (r) into normally distributed z-transformed correlation coefficients, which generates a 24 × 24 FC matrix for each participant. The individual connectivity matrices were averaged and converted into a binary matrix by applying a predefined threshold, T, such that connectivity coefficients larger than T were set to 1 and all others were set to 0. In the present study, 0.2 was chosen from a range of sparsity 0.1 to 0.5 (interval = 0.01) to obtain the binarized matrix. The small-world properties of the FC networks were analyzed for each threshold, including 8 global small-world parameters [clustering coefficient (C_p_), characteristic path length (L_p_), normalized clustering coefficient (γ), normalized characteristic path length (λ), global efficiency (E_global_), local efficiency (E_local_), hierarchy (β), and modularity (Q)], and 2 nodal parameters [nodal degree (k_nod_) and nodal efficiency (E_nod_)]. These 2 nodal parameters indicate the regional capacity of information communication in the brain network. A node was defined as a hub if the nodal parameters were two standard deviations greater than mean values (hub > mean_node ± 2SD) of all other nodes in the network.

### 2.5. Statistical analysis

Statistical analysis was conducted using IBM SPSS (version 26, Chicago, IL, United States). The normality of the data was analyzed by the Shapiro–Wilk test. Paired t-test was conducted to compare the correlation coefficients (Pearson correlation coefficients) and network topological properties (C_p_, L_p_, E_global_, E_local_, K_nod,_ and E_no_d) between resting state and Tai Chi standing meditation state. The significance level was set at *p* < 0.05. Data are presented as mean ± SD. Adjustments for multiple comparisons were performed by using Bonferroni correction.

## 3. Results

### 3.1. Cortical activation

It has been generally agreed that the HbO_2_ signal has a higher signal-to-noise ratio, whereas the HbR signal is more specific to the activation area (Huppert et al., 2006). Therefore, the activation maps of both HbO_2_ and HbR were calculated. One contrast T-map per participant, containing the resting state vs. the meditation state, was computed from both the HbO_2_ and HbR responses by applying interpolation to the channel-wise contrast values by the GLM method. The statistical significance in the group level was then determined by random effect analysis. The brain activation maps during Tai Chi standing meditation were shown in Figure 2. Compared to the resting state, significant increases in HbO_2_ concentration were observed in bilateral DLPFC, VLPFC, FEF and PMC (T = 1.71, *p* < 0.05, expected Euler corrected) as shown in Figure 2B. Meanwhile, significant decreases in HbR concentration were observed in left VLPFC, right PMC and DLPFC (T = 1.72, *p* < 0.05, expected Euler corrected) as shown in Figure 2C during Tai Chi standing meditation.

### 3.2. Functional brain networks

**Figure 3:**
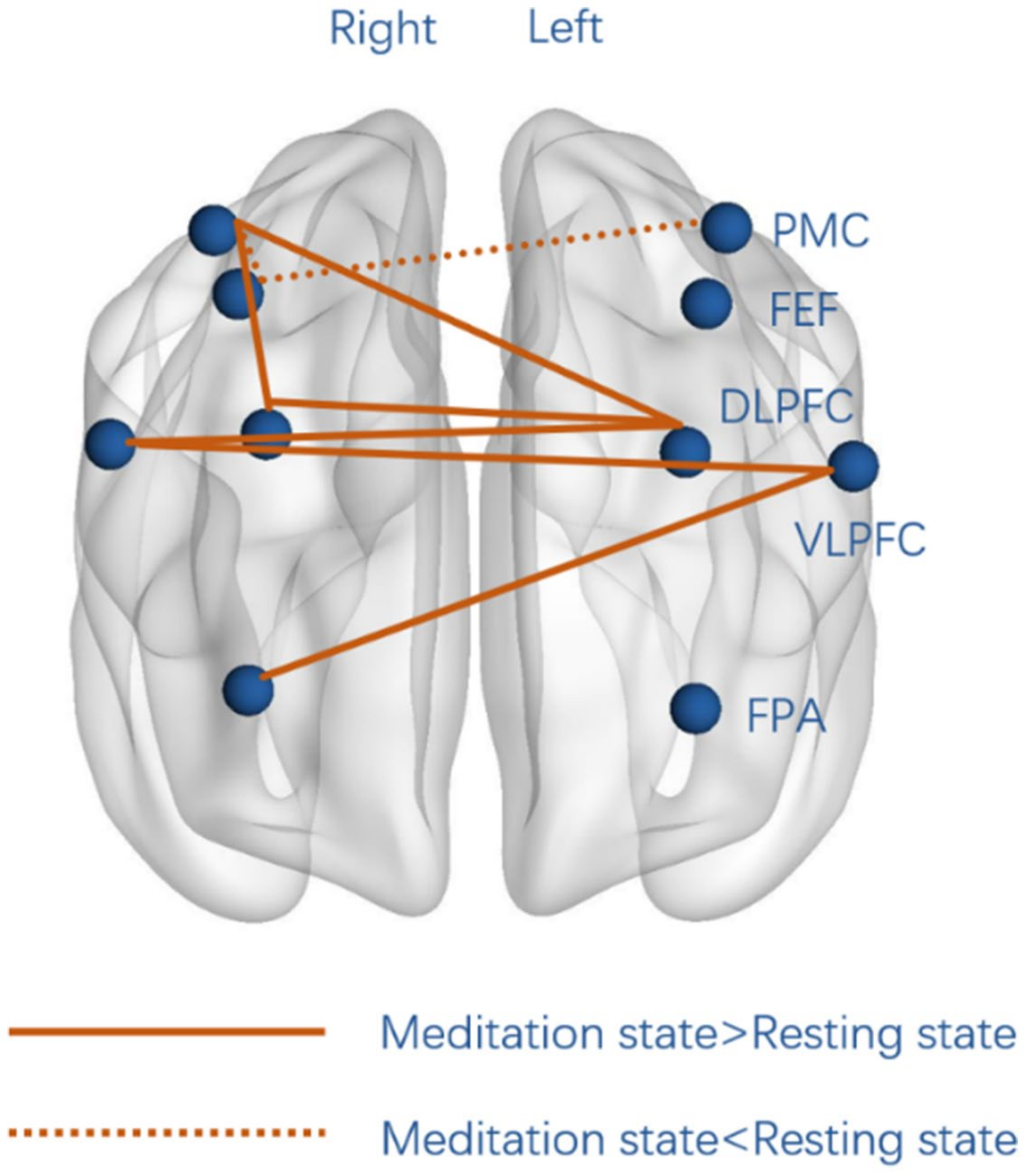
Significant differences in functional connectivity between standing mediation and standing rest. Full lines represent significantly higher functional connectivity during Tai Chi standing meditation than during standing rest, whereas dotted lines represent significantly stronger connectivity during standing rest than standing meditation. FPA, frontopolar area; DLPFC, dorsolateral prefrontal cortex; VLPFC, ventrolateral prefrontal; FEF, frontal eye field; PMC, pre-motor cortex.

Compared to the resting state, the meditation state exhibited significantly increased connectivity. In particular, left DLPFC showed significantly increased connectivity to the right DLPFC (*p* < 0.001), right VLPFC (*p* < 0.01), and right PMC (*p* < 0.01), while left VLPFC showed significantly increased connectivity to the right VLPFC (*p* < 0.01) and right FPA (*p* < 0.001) during Tai Chi standing meditation compared to standing rest (Figure 3). In addition, right DLPFC showed significantly higher connectivity to right PMC (*p* < 0.01) during Tai Chi standing meditation than during standing rest.

**Figure 4:**
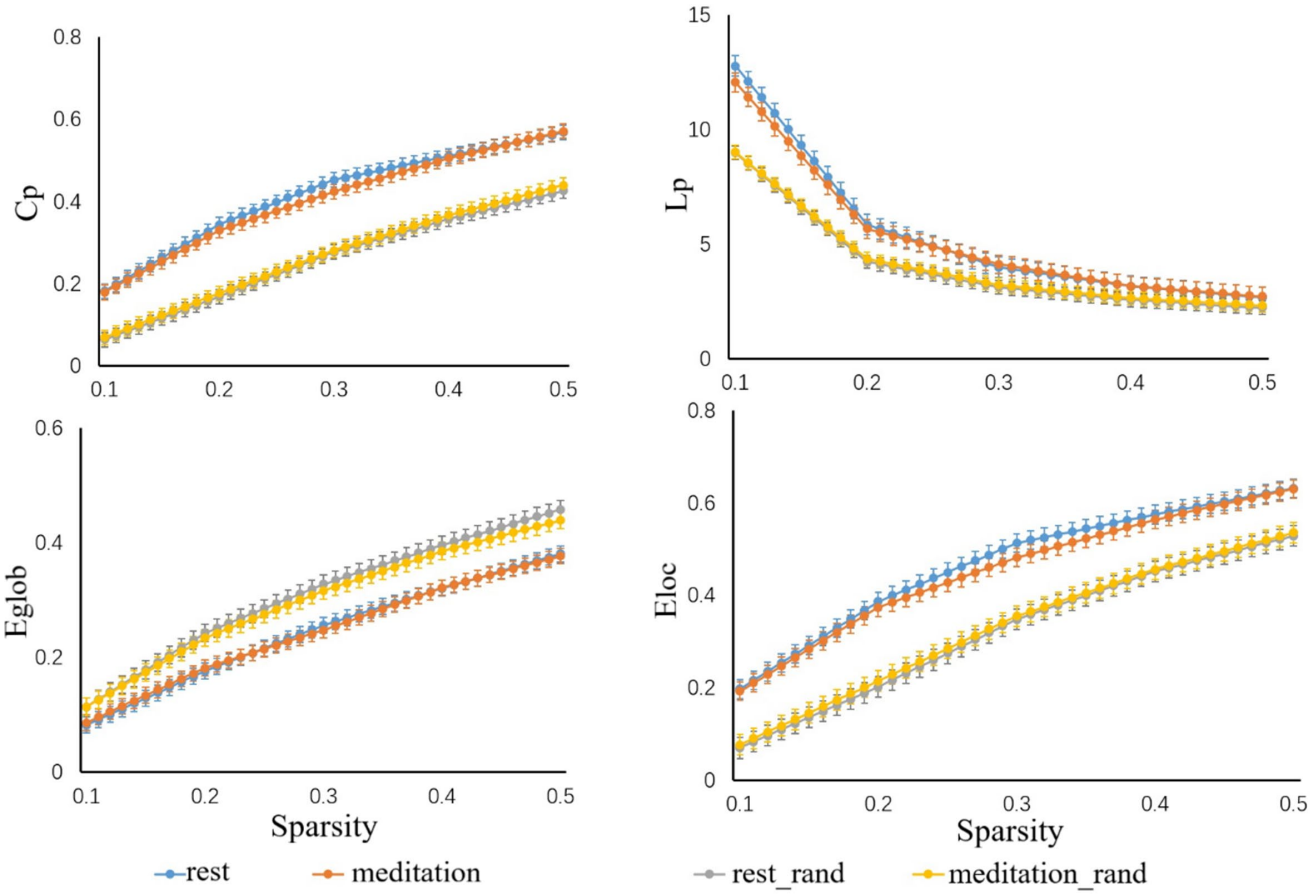
Small world properties in the threshold range of 0.10 < sparsity < 0.50. All participants showed higher C_p_, L_p_, and E_loc_ values, while their E_glob_ values remained relatively consistent when compared to 1,000 randomly generated networks.

Both the resting state and meditation state networks showed small-world properties in the threshold range of 0.10 < sparsity < 0.50 (Figure 4). Furthermore, all the networks exhibited higherγvalues, λ ≈ 1, and the σ of each network was larger than 1.1 for all participants in the entire threshold range of 0.10 < sparsity < 0.50 (Table 2). However, paired t-test showed no significant differences in the topological parameters (C_p_, L_p_, E_local_, E_global_) between the resting state and Tai Chi meditation state.

**Table 2:** Small world properties of the resting state and the standing meditation state.

Parameters	γ	λ	σ	C_p_	L_p_	E_local_	E_global_
Resting	2.68 ± 0.79	1.26 ± 0.26	1.28 ± 0.30	0.84 ± 0.08	2.04 ± 0.82	0.94 ± 0.09	0.49 ± 0.05
Meditation	2.72 ± 0.44	1.25 ± 0.25	1.30 ± 0.29	0.82 ± 0.08	1.91 ± 0.94	0.91 ± 0.10	0.49 ± 0.06

**Figure 5:**
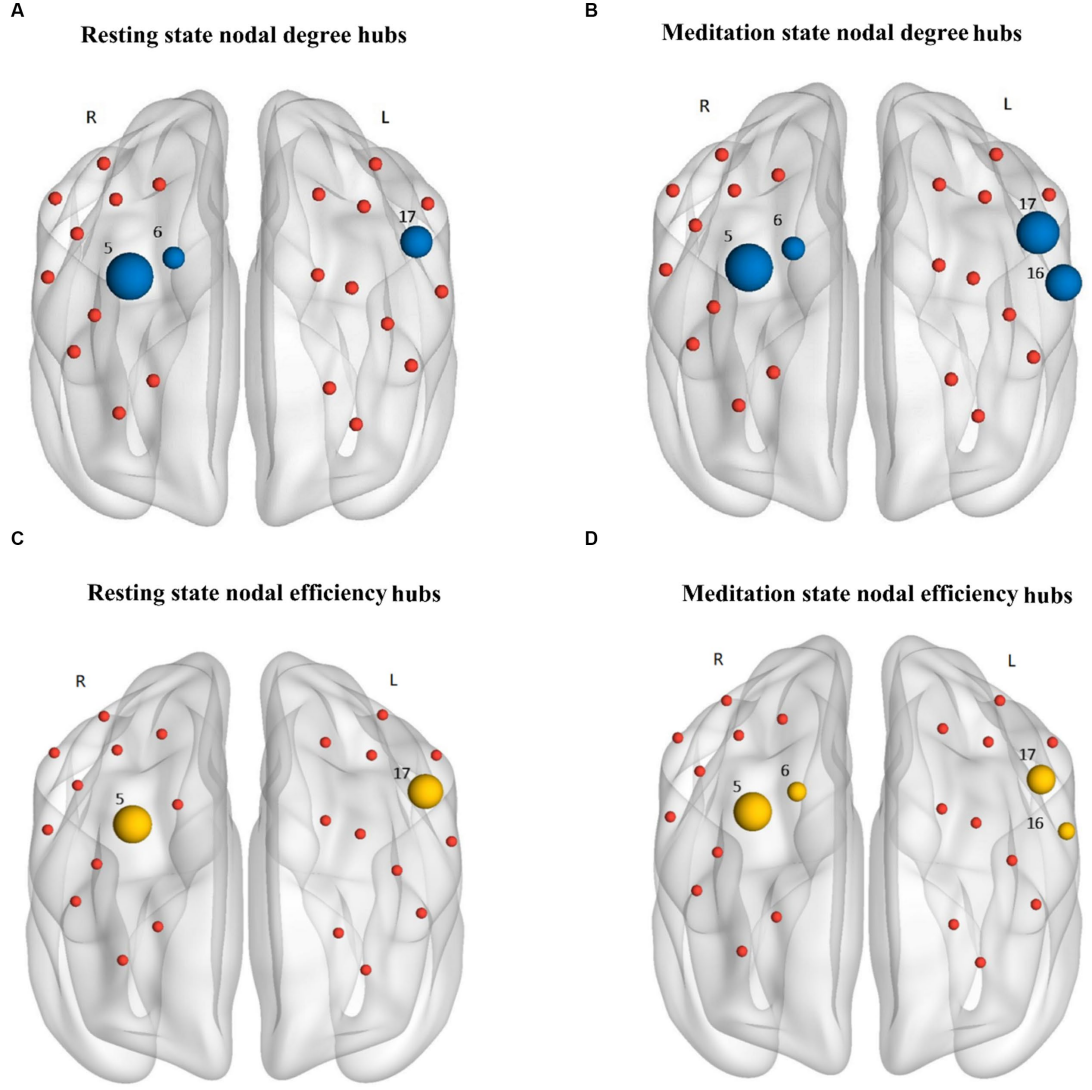
The nodal degree hubs (blue nodes) and nodal efficiency hubs (yellow nodes) in the HbO_2_ based functional networks of the resting state (**A,C**, respectively) and Tai Chi meditation state (**B,D**, respectively). A node was considered as a hub if the nodal degree was 2 standard deviations greater than mean degree of all the nodes in the network. The size of the hubs was normalized to the corresponding mean for all nodes in the network. The nodal degree hubs were located in left DLPFC (node 5 and 6), right DLPFC (node 17), and right VLPFC (node 16), while the nodal efficiency hubs were located in left DLPFC (node 5 and 6), right DLPFC (node 17), and right VLPFC (node 16). The red nodes only indicate the position of the nodes in the networks.

By sorting the nodal degree (K_nod_), 3 hubs [node 5 (left DLPFC), node 6 (left DLPFC), and node 17 (right DLPFC)] were identified during the resting state (Figure 5A), whereas 4 hubs [node 5 (left DLPFC), node 6 (left VLPFC), node 16 (right DLPFC), and node 17 (right DLPFC)] were identified during Tai Chi meditation state (Figure 5B). Node 16 (left VPPFC) became more strongly connected in the meditation state than during the resting state. For the nodal efficiency, 2 hubs (node 5, and node 17) were identified during the resting state (Figure 5C), whereas 4 hubs (node 5, node 6, node 16, and node 17) were identified during the Tai Chi meditation state (Figure 5D). Node 6 (right DLPFC) and node 16 (left VLPFC) became more efficiently connected in the meditation state than in the resting state. A significantly higher nodal efficiency (E_nod_) was also found in node16 (left VLPFC, *p* < 0.05) during standing meditation than during standing rest.

## 4. Discussion

In the present study, we used the within-subject design to investigate the prefrontal cortex hemodynamics and functional network changes during Tai Chi standing meditation compared to the standing rest in the same participants using the fNIRS technique. The meditation-related changes in PFC cortical hemodynamics and network organizations at both global and nodal levels of network organization were analyzed. The major findings are: (1) significant increases in HbO_2_ concentration were found in bilateral DLPFC, VLPFC, FEF and PMC during Tai Chi standing meditation. Meanwhile, significant decreases in HbR concentration were observed in left VLPFC, right PMC and DLPFC during Tai Chi standing meditation than during standing rest. (2) Compared to standing rest, we detected a significantly increased functional connectivity between the left and right PFC during Tai Chi standing meditation. (3) Both the resting and the Tai Chi meditation state functional brain networks exhibited small world architecture. Although the global topological properties of the prefrontal networks were conserved, more nodal degree and nodal efficiency hubs in DLPFC and VLPFC were identified during Tai Chi standing meditation than during standing rest.

### 4.1. Cerebral hemodynamic responses

According to the neurovascular coupling theory, there is a higher concentration of HbO_2_ and a decreased concentration of HbR in the activated brain regions, because the local supply of oxygen is greater than its consumption (Pinti et al., 2020). Since the HbO_2_ signals offer a higher signal-to-noise ratio (Huppert et al., 2006), the HbO_2_ hemodynamic responses during the meditation state are more broadly distributed than the HbR responses. The significantly increased HbO_2_ concentration in bilateral PFC during Tai Chi standing meditation is consistent with previous studies on meditation using fNIRS (Zheng et al., 2019), SPECT (Newberg et al., 2001), and fMRI (Tomasino and Fabbro, 2016), which showed significant increases of blood flow in the PFC. From a perspective of traditional Chinese medicine, Tai Chi standing meditation can be seen as reflecting optimized energy within the body and improved physical functions.

Previous studies have suggested that the decrease in HbR concentration reflects the cortical activation (Haeussinger et al., 2014). Significant decreases in HbR concentration were observed in left VLPFC, right PMC, and right DLPFC during Tai Chi standing meditation. The DLPFC, VLPFC, and PMC have been suggested as key regions of the attentional network in a growing body of literature (Ramirez-Barrantes et al., 2019). Recent meditation studies have shown that these brain regions (DLFPC, VLPFC, and PMC) are involved in sustaining and monitoring the focus of attention (Taren et al., 2017; Bauer et al., 2020). The activation of DLFPC during meditation has been reported in extensive studies, including both focused attention meditation (Brefczynski-Lewis et al., 2007; Hasenkamp et al., 2012) and open monitoring meditation (Farb et al., 2007).

### 4.2. Functional connectivity

When comparing the correlation coefficients in the meditation state with that of the resting state directly, we observed bilateral DLPFC, and bilateral VLPFC had increased functional connectivity in the meditation state. These findings are consistent with previous studies of functional connectivity among PFC sub-regions increases during meditation (Luders et al., 2011; Taren et al., 2017). The purposeful focusing of attention is considered crucial in all meditation practices. Tai Chi standing meditation focuses on bodily sensations (breathing, balance etc.) and one’s internal energy. Bilateral connectivity appears to be more characteristic of the Tai Chi standing meditation state in the present study, in particular, the increased functional connectivity between DLPFC and regions that comprise dorsal and ventral attention networks (VLPFC, FPA, and PMC). This may imply a neural mechanism underlying the enhanced attention and executive control observe during Tai Chi standing meditation. Previous studies have reported increased interhemispheric connectivity either during meditation or in long-term experienced meditators at resting state (Cahn and Polich, 2006; Luders et al., 2012; Bob et al., 2013). In contrast, the functional connectivity of the PMC and FEF increased significantly during the resting state as compared to the meditation state. FEF and PMC were thought to be associated with spatial attention focused on the visual environment (Szczepanski et al., 2013). Since the participants were standing still with their eyes gently closed during the Tai Chi meditation practice, these changes may be associated with a greater inward focus and withdrawal from the external world during Tai Chi standing meditation.

### 4.3. The topological organization of brain functional networks

Small-worldness has been considered as a universal principal for functional wiring of the human brain, which is organized to minimize wiring costs while maximizing the efficiency of information processing (Bassett and Bullmore, 2006). Previous resting state fNIRS studies have shown small-worldness in the functional brain networks in both young and older adults (Li et al., 2018). No significant difference was found in the small world properties (C_p_, L_p_, E_local_, E_global_) between the meditation state and the resting state in the present study. Tai Chi standing meditation, as a form of mindfulness training, is intended to enhance the ability to maintain a focus on the present moment. For Tai Chi Quan practitioners the characteristics of their small-world brain networks they have developed during years of practice appear to carry over into the resting periods when active meditation has ceased. Their mindful focus on the immediate present seems to continue by default.

Control of the flow of information is facilitated through matrix hubs that are critical gateways for the integration of diverse information sources. These hubs also help to minimize wiring and metabolic costs by providing a limited number of long-distance connections that integrate local networks. DLPFC and VLPFC become more central and more efficient in the brain network configuration during Tai Chi standing meditation state than standing rest. The VLPFC hub is a region frequently co-activated with DLPFC for emotional and attentional tasks (Lewis et al., 2005). Several meditation studies have suggested that mindfulness meditation enhances the ability to regulate emotion (Chambers et al., 2009; Farb et al., 2010). As reported in previous studies that Tai Chi practices can facilitate a positive effect on general outlook on life and foster a sense of calm and peace of mind (Sungkarat et al., 2018; Wu et al., 2019). Increased nodal degree and efficiency of bilateral DLPFC and lVLPFC during the Tai Chi standing meditation practice may strengthen the control of attention and emotion.

### 4.4. Study limitations

Some limitations of the present study should be mentioned. Firstly, we recruited male participants for the present study, our findings may not necessarily be extended to populations other than male Chen school Tai Chi Quan practitioners. Our test design, with measurements of Tai Chi standing meditation and standing rest among regular Tai Chi Quan practitioners, leaves the question unanswered what long-term effects Tai Chi meditation might have on cerebral hemodynamics and brain functional networks. Future studies are required to investigate the potential benefits of Tai Chi on brain oxygenation and connectivity in both healthy individuals and those with neurological conditions such as stroke, traumatic brain injury, and headaches (Mofatteh, 2021a). While the opportunities of artificial intelligence (AI) and machine learning in healthcare are promising, the AI-based prediction model (AIPM) can be used to predict patient outcomes for individuals with healthy and diseased brains (Mofatteh, 2021b; Chen et al., 2022). Secondly, the present findings of enhanced PFC hemodynamic responses and functional network organization indicate that individuals’ PFC functions are not the same in the meditation state as in the resting state, employing a within-subject design. Future investigations should extend this inquiry by examining the disparities in PFC hemodynamics and functional network dynamics between novice and experienced practitioners. Thirdly, fNIRS is limited in its penetration depth and thus less suitable for investigating deeper prefrontal cortical structures, such as the amygdala.

## 5. Conclusion

In the present study, the prefrontal cortex hemodynamics and functional network changes during Tai Chi standing meditation compared to the standing rest in the same participants were examined using the fNIRS technique. The general linear model and graph theory analysis were used to investigate PFC activity and functional networks. Our results demonstrated heightened neural activity, increased functional connectivity, and enhanced nodal efficiency in the DLPFC and VLPFC during Tai Chi standing meditation compared to standing rest. These findings suggest that Tai Chi standing meditation may enhance the regulatory capabilities of the DLPFC and VLPFC, contributing to improved control over attention, emotion, and cognition through the strengthening of PFC brain networks.

## Data availability statement

The raw data supporting the conclusions of this article will be made available by the authors, without undue reservation.

## Ethics statement

The studies involving humans were approved by Dalian University of Technology Ethics Committee. The studies were conducted in accordance with the local legislation and institutional requirements. The participants provided their written informed consent to participate in this study. Written informed consent was obtained from the individual(s) for the publication of any identifiable images or data included in this article.

## Author contributions

LQ: Conceptualization, Funding acquisition, Methodology, Writing – original draft, Writing – review & editing. G-LW: Data curation, Writing – original draft. Z-HT: Data curation, Validation, Writing – original draft. SG: Data curation, Writing – original draft. S-YY: Conceptualization, Investigation, Software, Writing – review & editing. Y-LY: Conceptualization, Methodology, Writing – review & editing. L-QL: Conceptualization, Methodology, Supervision, Writing – review & editing. Y-ZL: Conceptualization, Funding acquisition, Methodology, Supervision, Writing – review & editing.
